# Supplementary material for: Ballistic edge states in Bismuth nanowires revealed by SQUID interferometry
Source: Nat Commun. 2017 Jul 5;8:15941. doi: 10.1038/ncomms15941 (PMC5504270; doi:10.1038/ncomms15941)
Supplement: Supplementary Information [file ncomms15941-s1.pdf]

Type of file: PDF
Size of file: 0 KB
Title of file for HTML: Supplementary Information
Description: Supplementary Figures, Supplementary Table, Supplementary Notes and Supplementary References

Type of file: PDF
Size of file: 0 KB
Title of file for HTML: Peer Review File
Description:

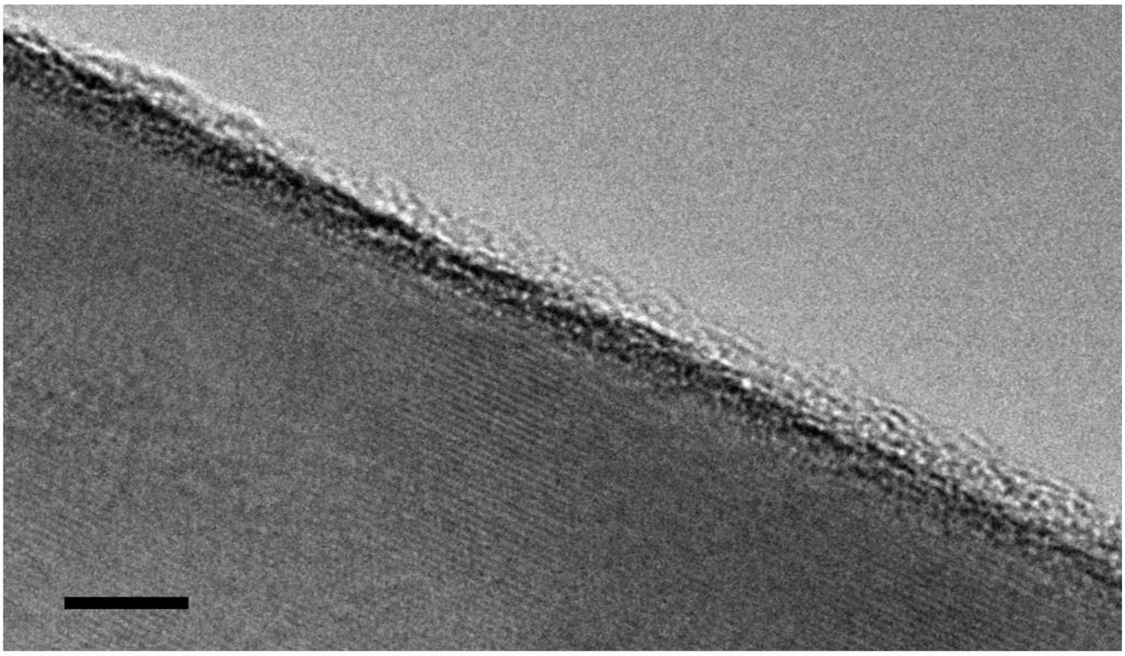

Supplementary Figure 1. **High resolution Transmission Electron Micrograph of a Bismuth nanowire**. The parallel atomic planes demonstrate the high cristallinity of the wire. A very thin amorphous oxide layer is also visible. Scale bar is 5 nm.

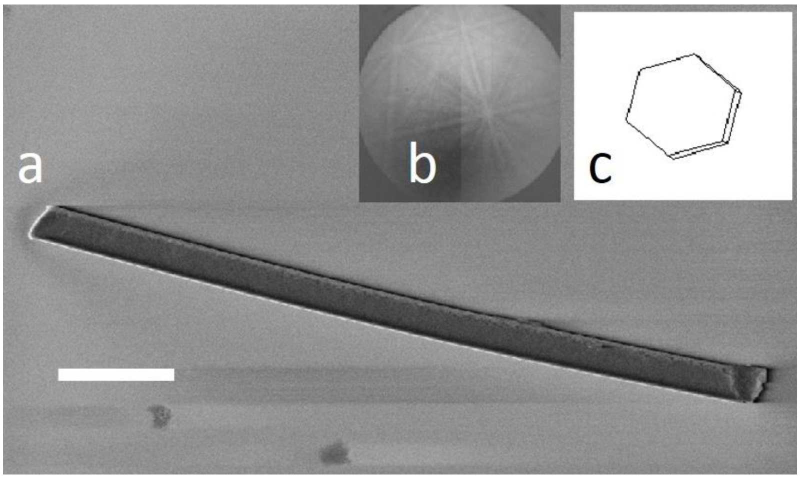

Supplementary Figure 2. **Bi nanowire with a top 111 facet. a** Scanning Electron micrograph of a Bi nanowire with a top (111) facet, as demonstrated by the Electron Backscattered Diffraction pattern in the form of Kikuchi lines (**b**) and the orientation of the reconstructed unit cell (**c**). Scale bar is one micrometer.

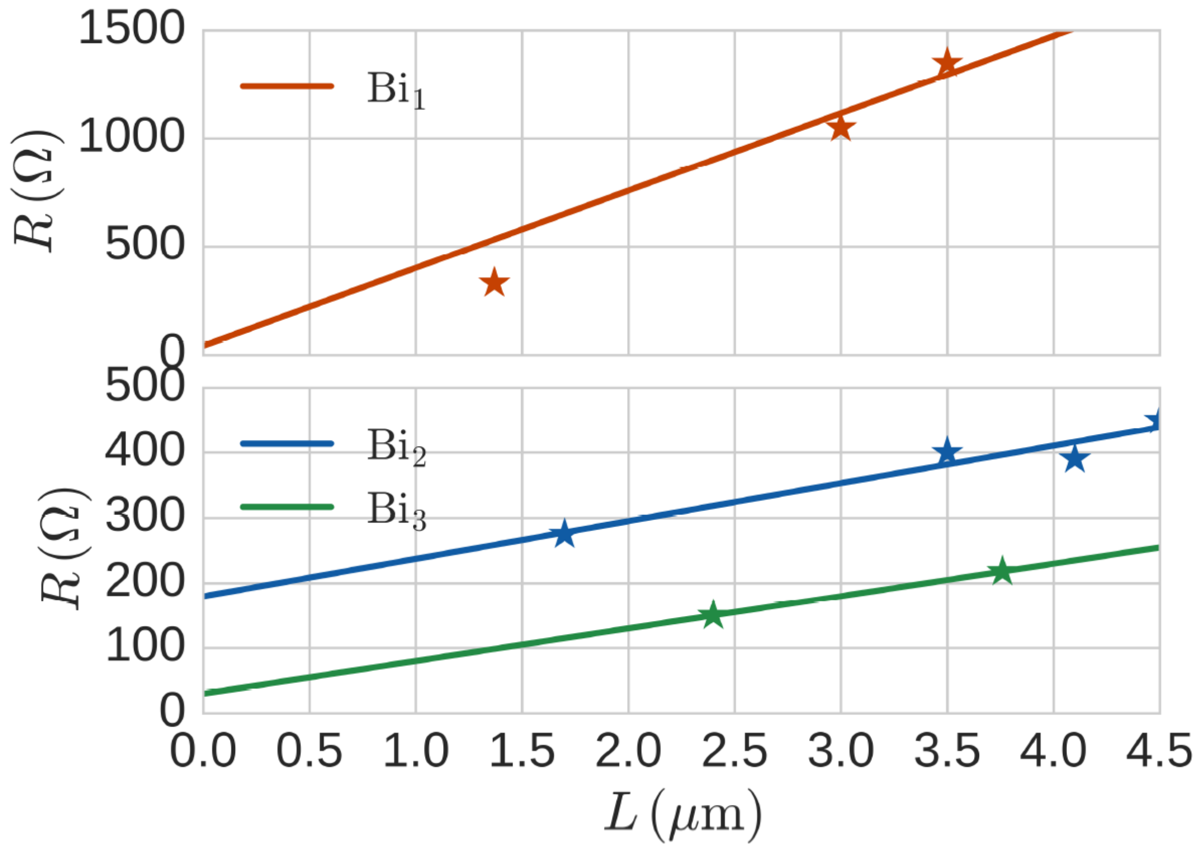

Supplementary Figure 3. **Length dependence of Normal state resistance of three Bi nanowires.**

We have measured several sections of three Bi nanowires. The sections are described in Supplementary Table 1. The length dependence of the normal state resistance displays a linear behavior, characteristic of a diffusive regime. Stars are measured resistance, solid line is linear fit with the constraint that the contact resistance is positive. The measurements were carried out at 4.2 K, except for section s_1JU. The resistance of that section was deduced from the resistance jump at the switching transition, which underestimates the resistance by the wire/superconductor contact resistance. Thus that point was not included for the fit. The slopes yield a resistance per unit length of 358 Ω, 57 Ω and 50 Ω μm^{-1} for Bi_1 Bi_2 and Bi_3 respectively. The dispersion between these values could be due to variations in wire diameters (which may vary by up to a factor of two).

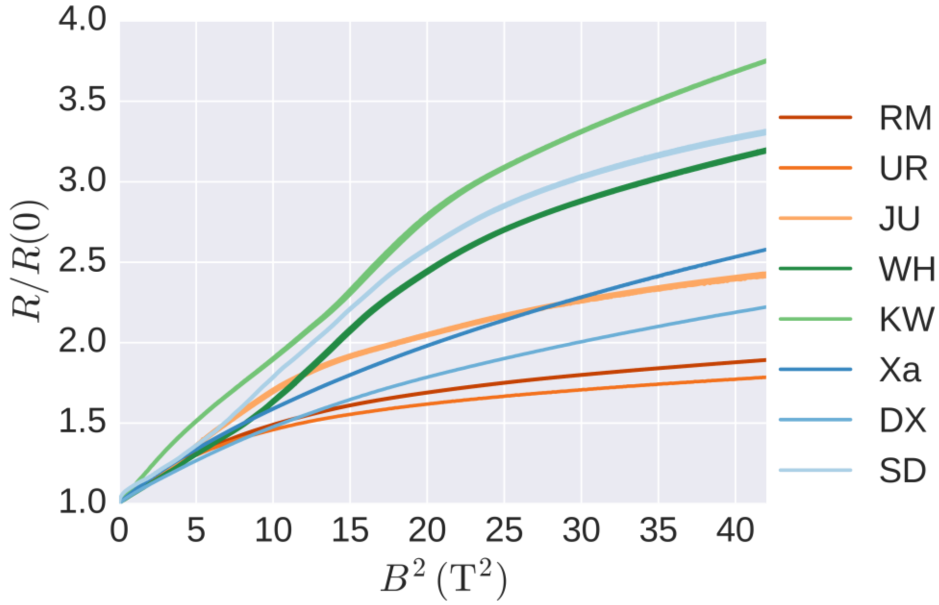

Supplementary Figure 4. **Magnetoresistances of eight segments of three Bi wires.** The magnetoresistances of the segments (described in Supplementary Table 1) are normalized by the zero field resistance and plotted against the square of the magnetic field B_z^2. A quadratic dependence on magnetic field is clearly visible on all samples at low fields, allowing the determination of the transport times given in Supplementary Table 1, and discussed in Supplementary Note 1. T=4 K.

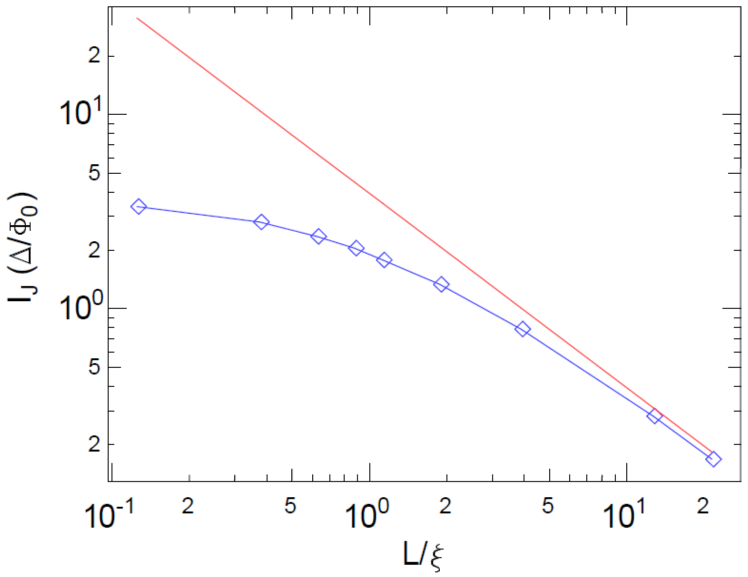

Supplementary Figure 5. **Simulation of critical current from short to long 1D ballistic SNS junctions.** Symbols display the calculated critical current amplitude I_J, in units of Δ/Φ_0, of ballistic one dimensional SNS junctions of varying lengths L, from the short to the long limit. The simulation uses a tight binding calculation described in (1) for a 1D periodic lattice. In the short junction limit $L<\xi$ we find $I_J = \Delta/\Phi_0$ and in the long junction limit the Josephson current is ev_F/L, where $v_F = 4\pi ta$ is the Fermi velocity and the superconducting coherence length $\xi = 2ta/\Delta$, a is the lattice spacing and t the hoping energy. The red line corresponds to the long junction limit ev_F/L.

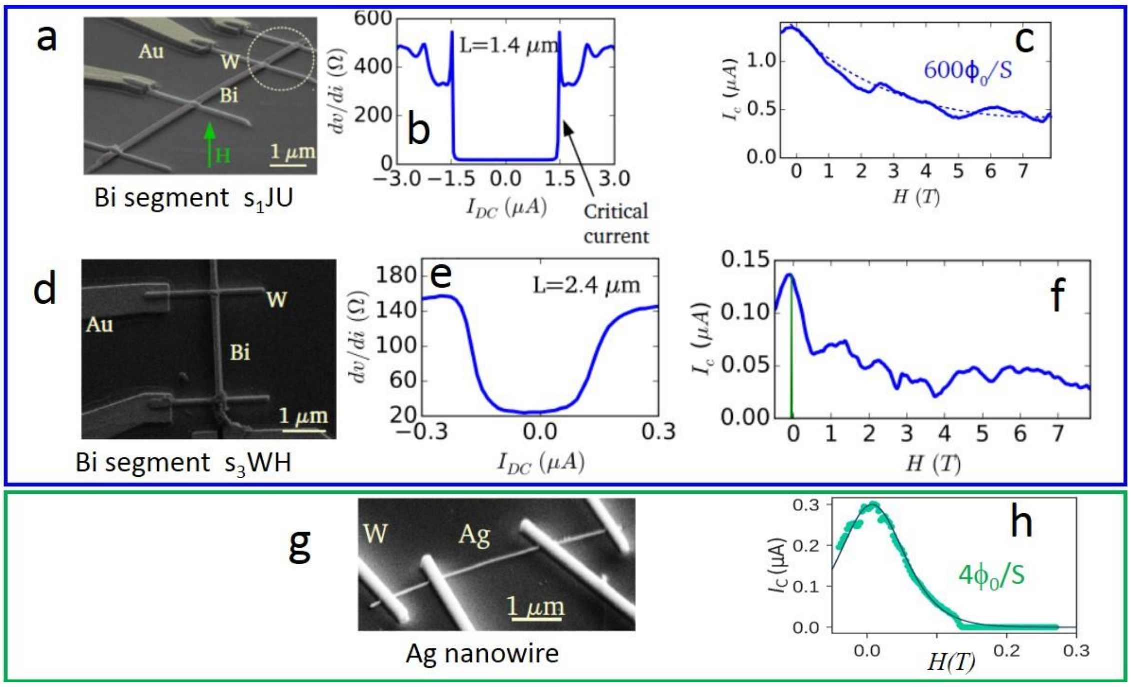

Supplementary Figure 6. **Comparison between field dependences of critical current of bismuth and silver nanowires**. Differential resistance versus current (panels **b** and **e**) and field dependence of the critical current (panels **c** and **f**) of two Bi nanowires (segments s_1JU and s_3WH, whose SEM images are presented in panels **a** and **d**) and one Ag nanowire (whose SEM image is presented in panel g and critical current versus field curve is plotted in panel **h**) of similar aspect ratio connected to the same type of superconducting tungsten electrodes. The critical current of the Bi nanowires extends beyond several hundred flux quanta threading the nanowires, whereas the critical current of the Ag nanowire decays with a typical scale of a few flux quanta threading the wire. This points to many channel diffusive transport in the Ag nanowire, and very few narrow channels in the Bi nanowires. The critical current of the two Bi nanowires sections also exhibit strong field modulations (not shown), similar to our previous findings in Bi wires (with unknown crystalline orientations (2)), that are the signature of a lateral confinement of the carriers.

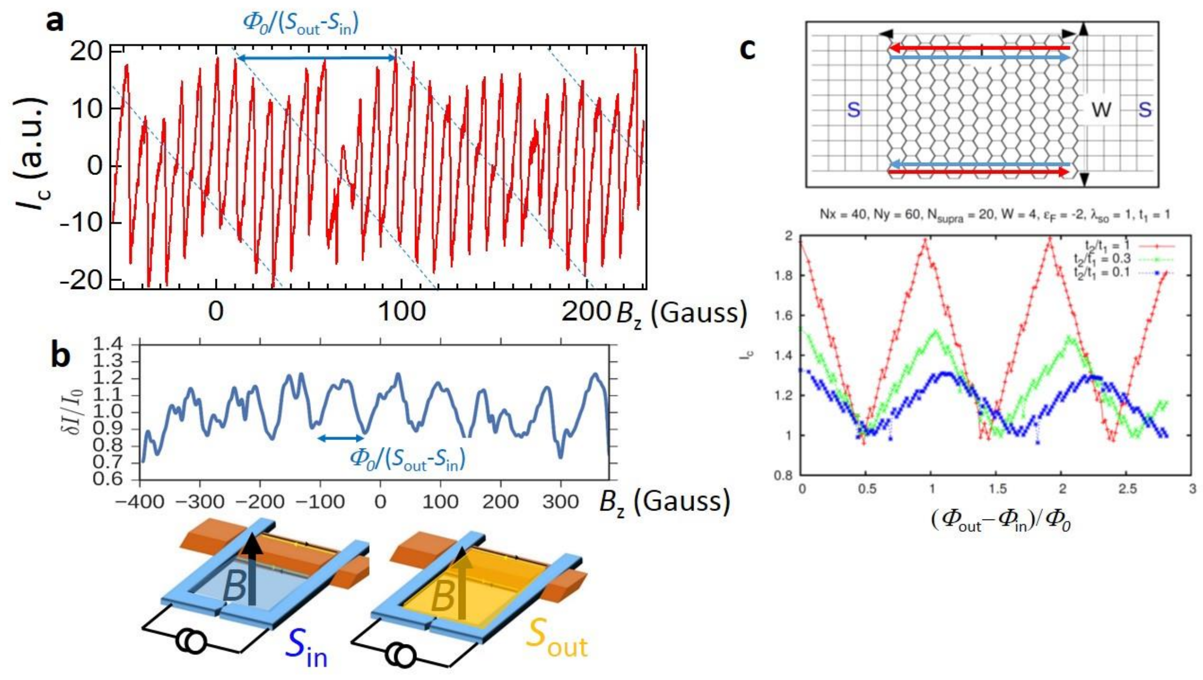

Supplementary Figure 7. **Periodicity of the critical current oscillations.** **a** High-pass filtered CPR of the experiment over several SQUID periods, displaying periodic modulation of the critical current with a main period of 9.5 Gauss, corresponding to one flux quantum through the nanowire area. An amplitude modulation due to beating of the two edge paths (of unequal transmission, see main text) is also visible, with a beat period of 85 Gauss (indicated by hatched lines), corresponding to a flux quantum through the nanowire. **b** Modulation amplitude extracted from the experiment by integration of the CPR over the main (9.5 Gauss) sawtooth period. Here as well, the period of one flux quantum through the wire appears. **c** Theory for the maximum supercurrent of two topological edge states with different transmissions t_1 and t_2.

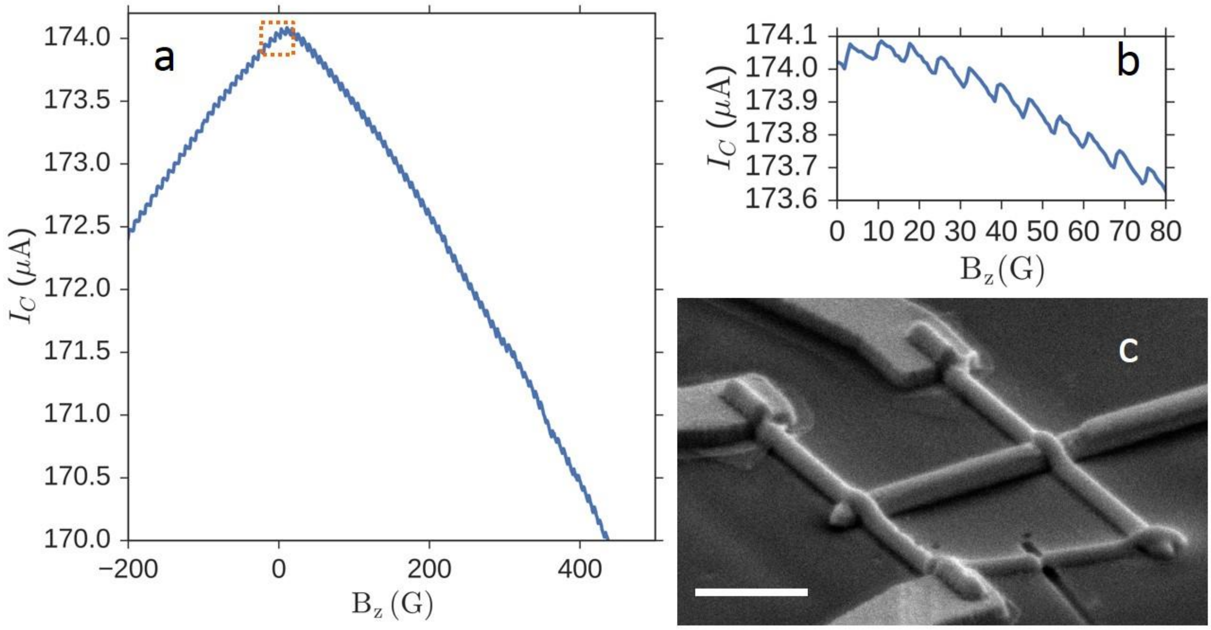

Supplementary Figure 8. **A second Bi(111) nanowire with a sawtooth current-phase relation.**

As in the device described in the main text, an asymmetric SQUID is fabricated with the focused-ion beam-assisted deposition of a superconducting W loop in parallel with a second Bi nanowire containing a (111) surface. A nanoconstriction is etched to generate the high critical current junction (See Scanning Electron Micrograph of Panel **c**). The asymmetry in this device is extremely large: the critical current of the W constriction was 173 µA and the modulation amplitude was 45 nA, as seen in panels a and b. Nevertheless, a periodic sawtooth signal in magnetic field with period of 8 G (corresponding to one Φ_0 in the area of the loop) is clearly superimposed on a slowly varying background, panels **a** and **b**. The second path cannot be identified in the CPR. This could be due to the difficulty to detect it in this extremely asymmetrical SQUID configuration, or more likely because the lower channel is not connected to the tungsten electrode, in contrast to the sample presented in the main text. The SEM of panel **c** indeed suggests that the W electrode may not contact the bottom nanowire facet. Scale bar is 1 micrometer.

Supplementary Table 1: **Characteristics of bismuth samples measured in Superconductor/Bi/Superconductor two wire configuration**. Nine sections of three different Bi nanowires, labeled s_1, s_2 and s_3, were investigated. Each sample corresponds to a different segment of the nanowire. Length is distance between superconducting contacts, R_N is the normal state resistance at low temperature, determined by the resistance jump above the critical current I_c for the three shortest segments, which display a supercurrent (s_1JU s_2SD and s_3WH). For longer segments that do not have a zero resistance at low bias, R_N is the resistance measured with a bias current of 1 μA. τ_{tr}/m^* is the transport time divided by the effective mass (in bare electron mass units), deduced from the magnetoresistance's parabolic dispersion shown in Supplementary Figure 4. R_N at RT is the sample resistance at room temperature.

Nanowire	Section	Length (μm)	R_N (Ω)	τ_{tr}/m^*(ps)	R_N at RT
Bi 1	s_1JU	1.4	330	18.6	693
	s_1UR	3.0	1050	17.5	1000
	s_1RM	3.5	1350	21.9	1100
Bi 2	s_2SD	1.7	275	19.0	478
	s_2DX	3.5	400	18.0	605
	s_2Xa	4.5	450	19.1	643
	s_2aZ	4.1	390	18.9	623
Bi 3	s_3KW	3.8	218	22.4	488
	s_3WH	2.4	150	22.8	500

Supplementary Note 1: Relative contributions from bulk and surfaces in the normal state

Transport in the normal state is due to bulk and surface states (in addition to topological edge states), and we now characterize the properties of both. ARPES experiments (3) yield a surface carrier density $n_s \approx 10^{17}$ m^{-2} and a corresponding Fermi wavelength $\lambda_s \approx 8$ nm. The effective mass is $m_s \approx 0.2\ m_e$ where m_e is the free electron mass. The band structure of bulk Bi is quite complex, leading to several types of carriers (3). In a nanowire, quantum confinement prohibits light electrons whose Fermi wavelength is greater than the wire's transverse dimensions. Thus, it is likely that the majority bulk states are hole states with an effective mass $m_b \approx 0.065\ m_e$ and a Fermi wavelength of $\lambda_b \approx 60$ nm. Those numbers suggest that in a 1 micron-long wire of width 200 nm and height 100 nm, the two non-topological surfaces contain roughly 100 times more carriers than the bulk.

Thus we can assume that the greatest contribution to normal state conductance comes from the surfaces, $G_N = G_s + G_b \sim G_s$. The mean free path on the surfaces is then deduced from the 300 Ω resistance of a 1 micron by 200 nm surface, via $\sigma_s = G_Q k_F l_s = GL/W = 1.7\ 10^{-2}\ \Omega^{-1}$, from which we deduce a surface-state mean free path $l_s \approx 300$ nm, and transport time $\tau_s \approx 3$ ps.

Interestingly, whereas the bulk states do not contribute much to the zero field conductance, we find that they yield an important contribution to the low field magnetoresistivity $\delta\rho(B)$ because their mobility $\mu_b \approx e\,\tau_b/m_b$ is greater than the mobility of the surface carriers, due to their smaller effective mass. Whereas elongated wires are not expected to exhibit sizable low field magnetoresistance, the situation is different when the conductivity $\sigma = Ne\mu$ results from 2 types of carriers with different mobilities (4):

$$\frac{\delta\rho(B)}{\rho} = B^2(\mu_b - \mu_s)^2 \frac{\sigma_b\sigma_s}{\sigma^2} \quad (1)$$

Since the surface conductivity is greater than the bulk conductivity, Supplementary Eq (1) becomes

$$\frac{\delta\rho(B)}{\rho} = e^2B^2\left(\frac{\tau_b}{m_b} - \frac{\tau_s}{m_s}\right)^2 \frac{\tau_b}{\tau_s}\frac{N_b}{N_s}\frac{m_s}{m_b} \quad (2).$$

The magnetoresistivity coefficient extracted from the plot of Supplementary Figure 4 is nearly sample independent, and yields:

$$\frac{\delta\rho(B)}{\rho} = (2\,ps)^2.\left(\frac{e}{m_e}\right)^2.B^2 \qquad (3)$$

So that we find $\tau_b \approx 2$ ps, and $l_b \approx 200$ nm. The bulk mobility μ_b is thus 2 times greater than the mobility of the surface states μ_s.

Supplementary Note 2: Field dependence of this and other (111) Bi nanowires' critical current

We have measured several sections of three different (111) nanowires. The sections are described in Supplementary Table 1. To induce superconductivity, we connect them to high-critical-field-superconducting electrodes one to a few micrometers apart. This leads to a sizable supercurrent at subKelvin temperature for the three shortest sections, whose length is of the order of or smaller than 2 μm. As shown in Supplementary Figure 5 for two samples s_1JU and s_3WH, we find that the critical current does not decay with magnetic field over the expected small range of one flux quantum through the wire's surface area, as in multichannel diffusive samples (5,6). Instead, the supercurrent persists up to unusually high fields, corresponding to several hundreds of flux quanta, persisting up to 10 Tesla in some samples. This is a clear indication of minimal orbital dephasing within Andreev pairs by the magnetic field, that is only possible if very few narrow conduction paths shuttle Andreev pairs across the wire, as suggested in previous experiments on Bi nanowires of unknown orientation (2). For comparison, we also show in Supplementary Figure 5 how the supercurrent induced in an Ag nanowire with the same aspect ratio as the Bi nanowire, connected to the same type of superconducting tungsten electrodes, is suppressed by a magnetic field above just a few Φ_0/S, where S is the wire area perpendicular to the field direction.

Supplementary Note 3: Self induction effects

It is well known that self induction effects can strongly distort the current phase relation in SQUIDS (7,8). In our experiments these effects were minimized by making the SQUID loops small enough. Screening of the flux into the loop of a SQUID is characterized by the parameter $\beta= 2\pi LI_c/\Phi_0$ where L is the inductance of the branch containing the small junction. This parameter is estimated to be respectively 0.1 and 0.06 for the DC SQUIDs containing the SIS and Bi nanowire-based Josephson junctions. These values of β yield small corrections on the SIS SQUID, and negligible ones for the Bi-based SQUID.

In practice, we have evaluated β by adding to the geometrical inductance the (much greater) kinetic inductance of the W wire. The kinetic inductance is related to the normal state resistance R_N via $L_K= R_N h/2\pi\Delta$ (9), yielding 30 pH per micrometer of the FIB-deposited W wires, and 1 pH per micrometer of Al wire. The inductance used of the SIS reference SQUID thus amounts to 200 pH, yielding β=0.1.

Supplementary Note 4: Interference between two ballistic paths as seen in the CPR and as calculated for a quantum spin Hall state with partial transmissions.

As mentioned in the main text, the supercurrent carried at the two (inner and outer) edges have a sawtooth-shaped CPR with slightly different periods, $\Delta B_{out}=\Phi_0/S_{out}$=8.5 G, and $\Delta B_{in}=\Phi_0/S_{in}$ =9.5 G. This leads to an amplitude and phase modulation (a beating) with a beat frequency given by the difference of the two frequencies $\Delta B^{-1}= \Delta B_{out}^{-1}- \Delta B_{in}^{-1}$, i.e. a period of 85 Gauss. This period corresponds to a flux quantum through the wire, and the factor of 10 between the SQUID period and the wire period corresponds to the ratio of the SQUID loop area over the wire facet area L_{sq}/W. The 85 G period is visible both in the form of the beating in the CPR shown in Supplementary Figure 7a and Supplementary Figure 7b, and (to within experimental uncertainly due to the position of the sample in the magnets in both experiments) in the period of the modulation of the critical current of the wire before insertion in the asymmetric SQUID configuration (fig 3 of main text). Finally, this 85 G also corresponds to the inverse of the difference of the frequencies between the main Fourier peak and its satellite, in the Fourier transform of the CPR shown in Fig. 2 of the main text.

We have also performed simulations on the hexagonal lattice with second neighbor spin-orbit interactions following the Kane and Mele model for the quantum spin Hall state (10). As expected when the Fermi energy lies in the spin-orbit gap, the Andreev spectrum of a ribbon connected to superconducting reservoirs consists of two spin-degenerate levels, crossing at the Fermi energy for a phase difference of π. They correspond to the counter-propagating 1D states along the edges of the ribbon. The maximum supercurrent, plotted in Supplementary Figure 7c, is found to oscillate with the flux through the ribbon with an amplitude which depends on the relative transmission at the NS interfaces along these two edges, and a period corresponding to one flux quantum through the wire.

Supplementary References

1. Ferrier, M., Dassonneville, B., Guéron, S. & Bouchiat, H., Phase-dependent Andreev spectrum in a diffusive SNS junction: Static and dynamic current response. *Phys. Rev. B* **88**, no. 17, 1–8 (2013).
2. Li, C. et al., Magnetic field resistant quantum interferences in bismuth nanowires based Josephson junctions. *Phys. Rev. B* **90**, 245427 (2014).
3. Hofmann, P., The surfaces of bismuth: Structural and electronic properties. *Prog Surf Sci*. **81(5),** 191-245 (2006).
4. Van Houten, H., Williamson, J. G., Broekaart, M. E. I., Foxon, C. T. & Harris, J. J., Magnetoresistance in a GaAs-AlxGa1-xAs heterostructure with double subband occupancy. *Phys. Rev. B* **37,** 2756–2758 (1988).
5. Cuevas, J. C. & Bergeret, F. S., Magnetic interference patterns and vortices in diffusive SNS junctions. *Phys Rev Lett*. **99**(21), 217002 (2007).
6. Chiodi, F. et al., Geometry-related magnetic interference patterns in long SNS Josephson junctions. *Phys. Rev. B* **86**, 064510 (2012).
7. Golubov, A. A., Kupriyanov, M.Y., Il'ichev, E., The current-phase relation in Josephson junctions. *Rev Mod Phys*. **76**(2),411-469 (2004).
8. Gaass, M. et al., Bistability in superconducting rings containing an inhomogeneous Josephson junction. *Phys. Rev. B* **77**, 024506 (2008).
9. Tinkham, M., Introduction to superconductivity, Mc-Graw-Hill (1996).
10. Murani, A., Chepelianskii, A., Guéron, S. & Bouchiat, H., Andreev spectrum with high spin-orbit interactions: revealing spin splitting and topologically protected crossings. Preprint at https://arxiv.org/abs/1611.03526 (2016).
